# An Overview of Recent Advancements in Microbial Polyhydroxyalkanoates (PHA) Production from Dark Fermentation Acidogenic Effluents: A Path to an Integrated Bio-Refinery

**DOI:** 10.3390/polym13244297

**Published:** 2021-12-08

**Authors:** Rijuta Ganesh Saratale, Si-Kyung Cho, Ganesh Dattatraya Saratale, Manu Kumar, Ram Naresh Bharagava, Sunita Varjani, Avinash A. Kadam, Gajanan S. Ghodake, Ramasubba Reddy Palem, Sikandar I. Mulla, Dong-Su Kim, Han-Seung Shin

**Affiliations:** 1 Research Institute of Biotechnology and Medical Converged Science, Dongguk University-Seoul, Ilsandong-gu, Goyang-si 10326, Gyeonggido, Korea; rijutaganesh@gmail.com (R.G.S.); avikadam2010@gmail.com (A.A.K.); 2 Department of Biological and Environmental Science, Dongguk University, Ilsandong-gu, Goyang-si 10326, Gyonggido, Korea; sk.cho@dongguk.edu (S.-K.C.); ghodakegs@gmail.com (G.S.G.); 3 Department of Food Science and Biotechnology, Dongguk University-Seoul, Ilsandong-gu, Goyang-si 10326, Gyeonggido, Korea; spartan@dongguk.edu; 4 Department of Life Science, Dongguk University-Seoul, 32 Dongguk-ro, Ilsandong-gu, Goyang-si 10326, Gyeonggi-do, Korea; manukumar007@gmail.com; 5 Department of Environmental Microbiology, School for Environmental Sciences Babasaheb Bhimrao Ambedkar University, Vidya Vihar 226 025, Uttar Pradesh, India; bharagavarnbbau11@gmail.com; 6 Gujarat Pollution Control Board, Gandhinagar 382 010, Gujarat, India; drsvs18@gmail.com; 7 Department of Medical Biotechnology, Dongguk University Biomedical, Campus 32, Seoul 10326, Korea; palemsubbareddy@gmail.com; 8 Department of Biochemistry, School of Applied Sciences, REVA University, Bangalore 560 064, India; sikandar.mulla@gmail.com; 9 Department of Environmental Science and Engineering, Ewha Womans University, Seoul 03760, Korea; dongsu@ewha.ac.kr

**Keywords:** dark fermentative hydrogen production, volatile fatty acids (VFAs), polyhydroxyalkanoates (PHA), biobased production, genetic engineering

## Abstract

Global energy consumption has been increasing in tandem with economic growth motivating researchers to focus on renewable energy sources. Dark fermentative hydrogen synthesis utilizing various biomass resources is a promising, less costly, and less energy-intensive bioprocess relative to other biohydrogen production routes. The generated acidogenic dark fermentative effluent [e.g., volatile fatty acids (VFAs)] has potential as a reliable and sustainable carbon substrate for polyhydroxyalkanoate (PHA) synthesis. PHA, an important alternative to petrochemical based polymers has attracted interest recently, owing to its biodegradability and biocompatibility. This review illustrates methods for the conversion of acidogenic effluents (VFAs), such as acetate, butyrate, propionate, lactate, valerate, and mixtures of VFAs, into the value-added compound PHA. In addition, the review provides a comprehensive update on research progress of VFAs to PHA conversion and related enhancement techniques including optimization of operational parameters, fermentation strategies, and genetic engineering approaches. Finally, potential bottlenecks and future directions for the conversion of VFAs to PHA are outlined. This review offers insights to researchers on an integrated biorefinery route for sustainable and cost-effective bioplastics production.

## 1. Introduction

**Figure 1:**
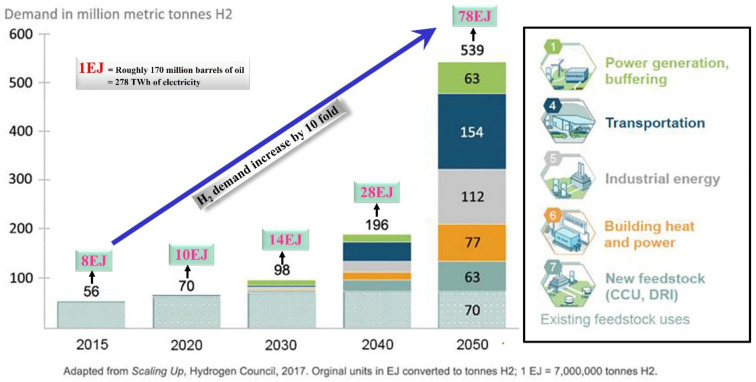
Appraisal of world-wide hydrogen demand from the year 2015 to 2050. Adapted from (Eljack et al., 2021; Kumar et al., 2021) [5,6].

The world population is anticipated to reach 10 billion in 2055 leading to increased exploitation and depletion of natural resources [1]. Economic development based on the extensive usage of fossil fuels, in sectors such as energy, food, and agriculture, presents unprecedented major challenges, including for fuel prices, combatting global climatic issues, and reducing fossil fuel dependency. According to the Department of Energy, U.S.A., it is predicted that there will be a transition from fossil-fuel-based transportation to a biomass-based fuel system by 2025 [2]. Renewable energy can address the primary problems associated with fossil energy, e.g., environmental sustainability and energy security. Amongst renewable energy sources, hydrogen is considered to be an ideal sustainable and eco-friendly alternative energy carrier for the future, by virtue of its high calorific value, and high gravimetric energy density (~33 kWh/Kg) [3,4]. Hydrogen energy technologies are used in many applications, such as heat and power generation, transportation, industrial raw materials extraction, and construction, thus generating substantial attention by scientists and industry. As a result of its societal, economic, and environmental advantages, worldwide hydrogen demand is constantly growing with a yearly growth rate of 6.07%, and is projected to increase by almost 80 EJ by 2050 [5,6], as illustrated in Figure 1.

Hydrogen can be produced using different technologies, including electrolysis, carbon capture, utilization and storage (CCUS), and biomass gasification. Conventional electrolysis and CCUS hydrogen production processes require large inputs of energy resulting from the burning of fossil fuels. Biohydrogen production, using a range of waste biomass resources, has attracted increased interest owing to its potential inexhaustibility, sustainable properties, and low energy requirements. Biohydrogen production processes can be categorized into dark fermentation, photo fermentation, and electrofermentation (MFC, MEC). Dark fermentative hydrogen generation using a wide variety of biomass resources (e.g., agricultural by-products, wastewater, food waste materials, municipal solid waste, biodiesel industry waste, etc.), under ambient temperature and pressure, has many advantages, including offering the most affordable and stable biohydrogen production, minimizing waste pollution, and being a carbon-neutral process [7,8].

**Figure 2:**
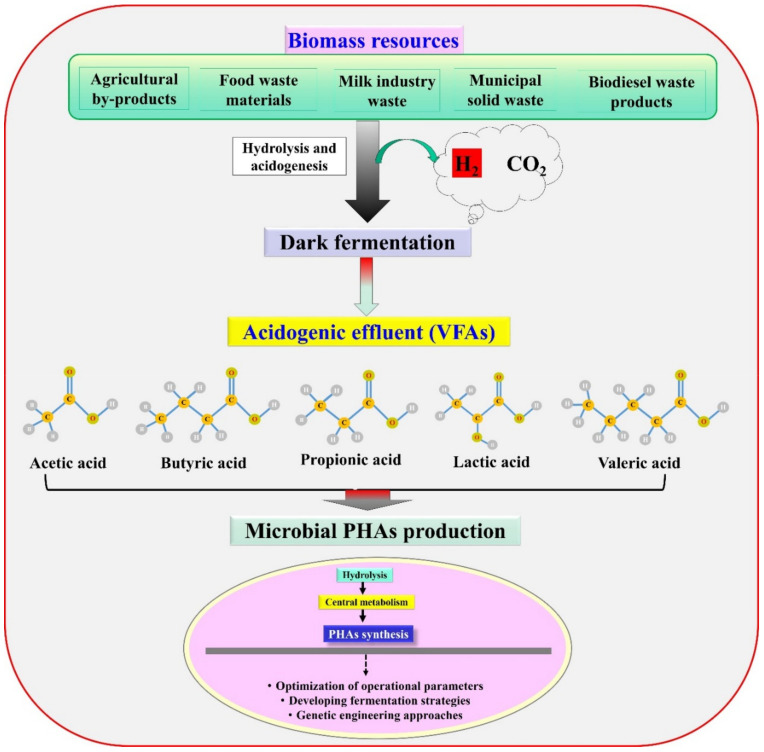
Schematic representation of utilizing dark fermentative acidogenic effluents (VFAs) for PHA production.

Despite many advantages, dark fermentative hydrogen production has many constraints for its commercialization. In particular, the substrate requires vigorous pretreatment and there is a low experimental yield. The low hydrogen yield is mainly due to the metabolic shift of the biocatalyst to intermediate metabolites/by-products and the consumption of H_2_ by homoacetogens or propionate-generating bacteria. The metabolic by-products, mainly VFAs, compete with metabolic routes available for H_2_ synthesis, and are not further transformable to H_2_ by fermentation, resulting in lower H_2_ yield [9,10]. VFAs remain a major unutilized carbon source in dark fermentation effluents; valorisation of this effluents into PHA would be a feasible alternative to enhance the energetic improvements and economic benefits of the procedure [9]. Currently, various researchers are examining integrated biorefinery routes for the efficient conversion of dark fermentative acidogenic effluents to generate PHA. This review article summarizes recent developments in VFAs to PHA production (Figure 2). The present review presents a summary of VFAs, their physicochemical properties, the microorganisms associated with inexpensive and sustainable PHA synthesis, and their metabolic pathways. The effects of operational parameters, various fermentation strategies for improved PHA production, and associated obstacles are also outlined. Finally, current challenges associated with the development of integrated VFAs to PHA biorefinery schemes, and future opportunities are discussed to enable the procedure to become sustainable and economically viable.

## 2. Chemical Properties and Market Potential of Acidogenic Effluent (VFAs)

**Figure 3:**
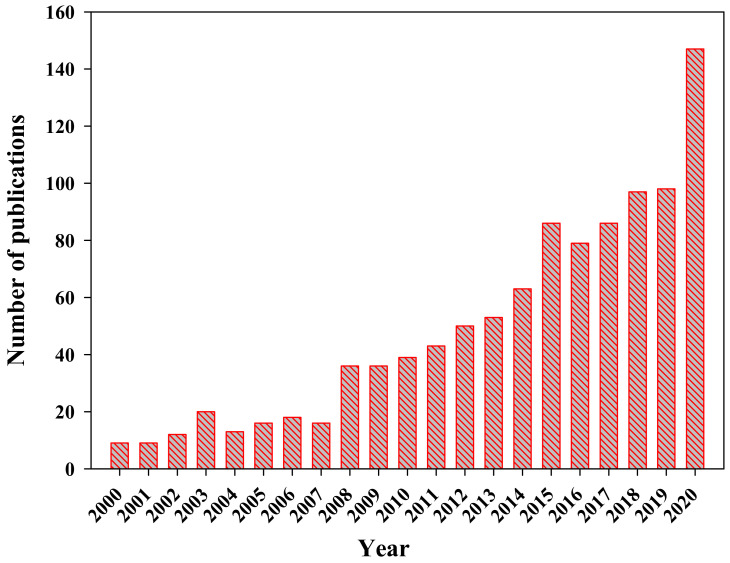
Publication record related to VFA production using anaerobic fermentation from the year 2000 to 2020 (Scopus—document search results).

After dark fermentation, the generated acidogenic effluents mostly comprise of volatile fatty acids which can serve as an inexpensive and sustainable substrate for bioplastics production. Across the planet, VFAs act as an important carbon source for microbial flora and are integral to organic carbon cycling [11]. VFAs are a renewable source and essential building block chemicals for the chemical manufacturing sector. Due to their numerous applications in the chemical manufacturing, tanning, food, beverages, pharmaceutical and cosmetic industries, their market value has greatly increased [12]. The chemical properties, total global market demand, and increasing compound annual growth rate (CAGR), are depicted in Table 1. VFA manufacture is usually by means of chemical methods, mainly the oxidation and carboxylation of aldehyde and alkenes. These processes require petrochemical sources which makes the process more energy-intensive and less eco-friendly [13]. According to the U.S. Department of Energy (DOE, Washington, DC, USA) VFAs are considered as “top value-added chemicals from biomass” [14]. Moreover, biomass-based VFA production using anaerobic digestion is receiving more attention. Current trends in publications associated with VFA generation using anaerobic digestion are depicted in Figure 3.

**Figure 4:**
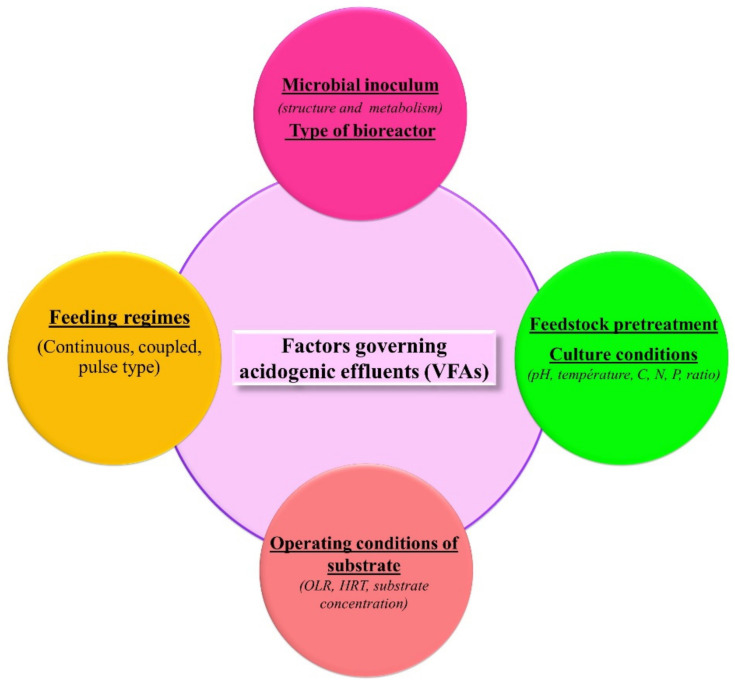
The details of factors influencing volatile fatty acid generation during acidogenesis by dark fermentation process.

Various factors, particularly operational parameters, including culture conditions, operating conditions of the substrate, and the feeding regime, affect production of VFAs by anaerobic digestion and these should be optimized. In addition, substrate pretreatment, and pretreatment of the microbial seed culture, have been found to be useful for effective acidogenesis and VFA production. To achieve competent microbial communities for hydrogen production and generation of VFAs, different pretreatment methods for the anaerobic inoculum, such as heat shock, chemical application, aeration, microwaves, and ultrasound have been thoroughly investigated [15,16]. Not only are process conditions, pretreatment methods, microbial structure and metabolism important in the production of VHAs, but also their concentration and chemical composition (Figure 4). Optimization of factors governing VFA yield is essential to enhance the composition of VFAs and their further conversion into PHA. Thus, an integrated VFAs-PHA biorefinery approach can make the process inexpensive, eco-friendly, and practically applicable.

## 3. Importance of PHA Relative to Synthetic Plastics

Polyhydroxyalkanoates (PHA) are precursors of bioplastics that have attracted research attention owing to their biodegradability, biocompatibility, and chemical diversity. The world-wide bio-plastics market shows tremendous growth potential in producing sustainable products for diverse applications in the biomedical, food packaging, electronics, automotive, and agricultural industrial sectors [21,22]. The details of known worldwide pilot and large-scale PHA production using various carbon sources are presented in Table 2. Universal plastic manufacture touched 359 million metric tons in 2018, compared to 200 million metric tons in 2002. Additionally, it is predicted that the bioplastic market value will touch 6.73 billion USD by 2025. It is anticipated that by 2025, the global PHA market will grow by about 14% and become one of the main sectors in the bioplastics market. However, the manufacturing cost of PHA (USD4000–15,000/Mt) is substantially greater compared to the cost of synthetic plastic manufacturing (USD1000–1500/Mt) [23]. The carbon source is one of the major obstacles in microbial synthesis of PHA which represents about 50% of the overall production cost. In consequence, extra focus is needed to develop PHA production using various low-cost substrates at commercial scale. Microorganisms, such as *Alcaligenes latus*, *Ralstonia eutropha*, *Azotobacter beijerinckii*, *Bacillus megaterium*, *Klebsiella* sp., *Pseudomonas* sp., *Lysinibacillus* sp., and some fungi and archaea, were found to accumulate the PHA molecule as a reserve food material under severe environmental conditions and nutritional inadequacy [22,24].

Various biomass resources including agricultural residues, molasses, whey, glycerol, waste oils have been considered for PHA production [25,26]. Recently, some investigators studied PHA production by employing CO_2_ as a potential substrate and used seawater as a selective medium to avoid the need for sterilization procedures to make the process more inexpensive and environmentally benign [27,28].

## 4. VFAs as a Potential and Inexpensive Substrate for PHA Production

Synthesis of PHA using fermentative acidogenic effluents (VFAs) makes the procedure more cost effective and beneficial to alleviate the environmental problems associated with generated effluents. Acidogenic effluent mainly consists of acetate, butyrate, propionate, and valerate and its composition can influence the types of PHA accumulation and characteristics. Variation in the constituents of VFAs conceivably occurs because of characteristics of the components of the organic complexes existing in biomass waste materials [29]. It has been observed that a higher concentration of even-numbered VFAs (e.g., acetic acid, butyric acid) increases the 3-hydroxybutyrate portion whereas, a higher concentration of odd-numbered VFAs (e.g., propionic acid, valeric acid) results in a higher fraction of 3-hydroxyvalerate in the synthesized PHA [30]. Recently, some investigators have utilized the VFAs generated in hydrogen-producing reactors for PHA production using mixed microbial cultures. By employing VFAs with 6 g/L concentration the resulting PHA consisted of approximately 95% P(3HB) and approximately 3% P(3HV). This variation in the composition of PHA occurred because the acidogenic effluents contained higher amounts of acetic acid and lower concentrations of butyrate and propionate [31]. In another study, two kinds of fermented cheese whey were employed as a potential carbon source for PHA production. The two sets had a different composition of VFAs, where the first set comprised lactate, acetate and butyrate acids in the proportions of 58/16/26 (%), respectively, whereas the other set comprised acetate, propionate, butyrate, lactate, and valerate in the proportions of 58/19/13/6/4 (%), respectively. The results showed that the first set produced 3-hydroxybutyrate, while the second set yielded 40% of 3-hydroxyvalerate and 60% of 3-hydroxybutyrate in the synthesized PHA [32]. Hong et al. [33] showed that after anaerobic fermentation of palm oil mill effluent the resulting acidogenic effluent was found to be a vital and inexpensive substrate for PHA synthesis by *Ralstonia eutropha* ATCC 17699 in which the significant yield of PHA recorded was approximately 11.4 g L^−1^. In another study, isolated *Comamonas* sp. EB172 showed an ability to produce significant quantity of copolymers P(3HB-co-3HV) using a mixture of VFAs [34]. Wang et al. [35] showed the potential of *Bacillus cereus* strain HY-3 for PHA production using acetate as a potential substrate in a high salinity medium. It was observed that acetic acid, where the concentrations were 0.5 and 5.0 g/L, showed the maximum 3 HB amount in the medium of approximately 41.0 ± 0.415% and 49.2 ± 1.21%, respectively.

Recently, Garcia-Gonzalez et al. [36] investigated the synthesis of homopolymer (PHB) and copolymer P(3HB-co-3HV) with acetic acid and CO_2_ using *C. necator* DSM 545. In another study, *R. eutropha* was cultivated in condensed corn solubles and the effects of individual fatty acids on PHA synthesis were evaluated. The results suggested that supplementation of butyric acid at a concentration of 5 g/L enhanced bacterial cell growth and PHA production [37]. Co-culture of potential microbial strains has been applied for the utilization of VFAs for PHA production. A combined microbial culture of *Pseudomonas* sp. and *Bacillus* sp. was utilized for PHA synthesis using VFAs and a pure carbon source (glucose). The results confirmed that application of microbial co-culture is an effective strategy for the assimilation of substrate and PHA production relative to pure culture [38]. Under the nitrogen-depriving conditions in artificial biogas-based cultivation, *Methylocystis hirsuta* produced approximately 52% PHA of DCW using acetate and butyrate as co-substrates [39]. Catalán et al. [40] utilized *Herbaspirillum seropedicae* Z69 strain to produce P (3HB-co-3HV) utilizing propionate as the potential substrate. A maximum yield of 0.80 g g^−1^ of 3HV was recorded which was substantially lower than the theoretical yield (1.35 g g^−1^). *Ralstonia eutropha* KCTC 2658 was assessed for PHA production using acetate, propionic, and butyric acids as a substrate. The results suggested that this strain showed the ability to synthesize a copolymer of P(3HB-co-3HV) under optimized conditions. It was suggested that the quantity of VFAs to co-substrates directly influences microbial cell growth, PHA content, and biopolymer composition [41]. Similarly, some investigators have shown that employing valerate as a co-substrate with acetate can produce effective copolymers with a higher contribution of HV of approximately 84.77 mole% [42]. Recently, Saratale et al. [43] examined the potential for use of *R. eutropha* for PHB production using chemically pre-treated wheat waste biomass. They observed that supplying 1 g/L acetate in the fermentation medium enhanced bacterial growth and PHB titer relative to the control set (without any supplementation). The authors suggested that acetate may act as an intermediate metabolite for the PHB production pathway through which there is increase in cell growth and PHB titer. Some investigators have determined the effect of supplementation of valerate on co-polymer production. The results indicated that addition of valerate to the fermentation medium induces both bacterial growth and biopolymer production. However, after 30 h of fermentation addition, a negative effect was observed because higher amounts of PHB accumulated in the bacterial cell [44]. Padovani et al. [45] appraised the capability of both blue-green and purple non-sulphur photosynthetic microbes for the synthesis of biopolymers via photo-fermentation. It was found that the total concentration of VFAs and their soluble chemical oxygen demand influenced PHA production. The results suggested that the composition of VFAs is significantly involved in determining the characteristics of accumulated PHA. A literature review of PHA production using individual and mixed VFAs by pure microbial culture is summarised in Table 3.

## 5. Metabolic Pathways Using Various VFAs

**Figure 5:**
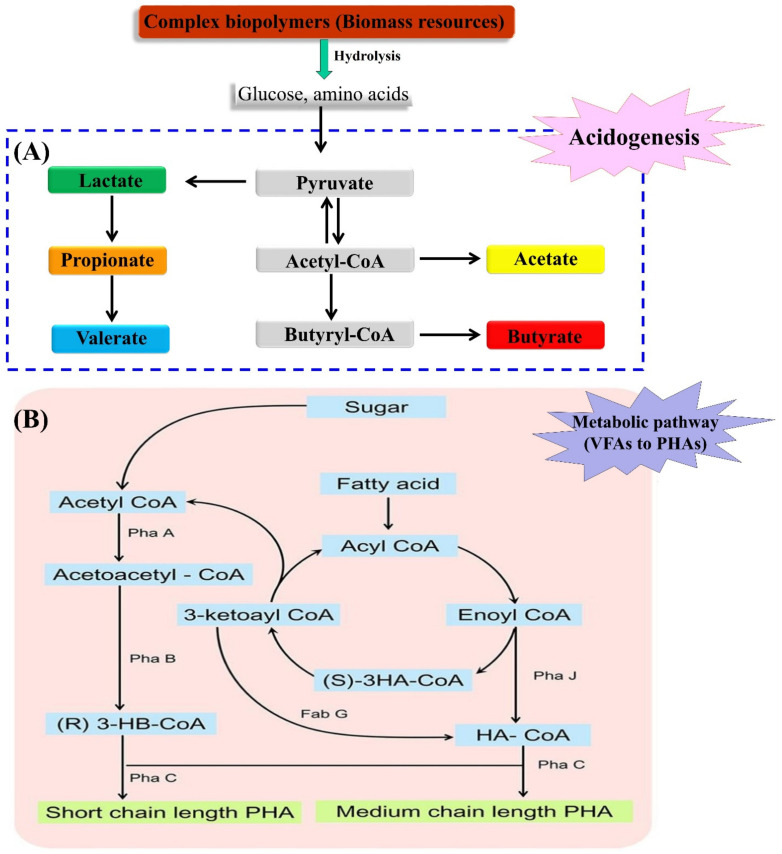
The synthesis mechanism of (**A**) volatile fatty acids during acidogenesis, and (**B**) the metabolic routes of VFAs to PHA production (adapted from Banu et al. [61]).

The synthetic mechanisms of volatile fatty acids during acidogenesis and the metabolic routes of VFAs to PHA production is shown in Figure 5. In acidogenic effluents the major component is acetic acid, which contributes approximately 30 to 80%, while other types of VFAs are found in scarce amounts. In acidogenic effluents, acetate can be considered the most favorable substrate for PHA production which is a three-step process. Firstly, acetic acid is transformed into acetyl-coA and combination of two molecules of acetyl-coA leads to the formation of acetoacetyl-CoA using the enzyme β-ketothiolase (phaA). The resulting acetoacetyl-CoA, facilitated by NADPH-dependent acetoacetyl-CoA reductase (phaB), is then transformed into (R)-3-hydroxybutyryl-CoA, and finally leads to the formation of PHA using the enzyme PHA synthase (phaC) [22,61].

During the anaerobic fermentation of carbohydrates, butyric acid is generated with higher production mainly when using *Clostridium* species. In general, PHA production, consisting of 3-hydroxubutyrate monomers, entails use of fatty acids as the potential substrate for the synthesis of short chain length (scl) or medium chain length (mcl) biopolymers (Figure 5B). By following the β-oxidation pathway, improvement in the effective transformation of VFAs to PHA was observed. During this transformation two major biocatalysts, 3-ketoacyl-CoA thiolase (FadA) and 3-hydroxyacyl-CoA dehydrogenase (FadB), are mainly involved. Whereas, during synthesis of mcl PHA exclusion of enzymes FadA and FadB takes place and the fatty acids are converted into 3-hydroxyacyl-CoA [62,63]. Propionic acid and valeric acid act as intermediates for the synthesis of 3HV in a larger proportion. In the presence of propionate, is changed into propionyl-coenzyme A and undergoes reaction using acetyl-coenzyme A to produce PHV as a final product. In addition, under anaerobic circumstances, PHV can be produced by the condensation of propionyl-CoA and valeryl-CoA which are produced from pyruvate and valerate, respectively [64].

## 6. Approaches to Enhance VFAs to PHA Synthesis

### 6.1. Optimization of Fermentation Process Parameters

**Figure 6:**
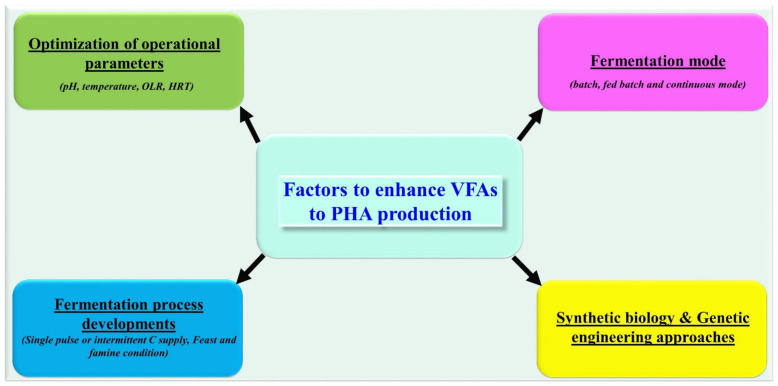
Details of various operational and advanced strategies to enhance VFAs to PHA production.

Optimization of fermentation process parameters are vital factors to achieve the effective transformation of VFAs into PHA. The appropriate concentration of VFAs in the fermentation medium is essential for the assimilation of VFAs and subsequently the enhancement of microbial growth and PHA yield. If the concentration of VFAs is too high, it leads to acidic conditions which reduces the proton gradient across the membrane and eventually effects their uptake, microbial growth, and PHA accumulation [65]. OLR plays a significant role in maintaining a substantial concentration of organic load during fermentation which directly influences PHA production. Recently, Amulya et al. [66] demonstrated the capacity of *Bacillus tequilensis* for synthesis of PHA using spent wash wastes as a substrate at diverse OLRs, such as 0.66, 1.32, 1.98, and 2.64 kg COD/m^3-day^. Of these, the maximum PHA accumulation and bacterial growth was observed at 1.32 kg COD/m^3-day^ OLR. However, further increase in OLR showed negative effects in terms of osmotic shock, bacterial cell growth, and PHA production. Similar results were recorded during PHA synthesis using *Serratia ureilytica* by employing acetate, propionate, and butyrate as a substrate at varying OLRs, with maximum cell growth and PHA accumulation (84%) recorded at OLR2 [67]. Moreover, HRT has also been shown to influence VFAs generation during the acidogenesis process of various complex substrates. Thus, VFAs concentration and operational conditions (e.g., pH, temperature, OLR, HRT, substrate, C:N ratio) need to be optimized for the effective assimilation of VFAs by biocatalysts and their further conversion into PHA (Figure 6).

### 6.2. Employing Various Fermentation Mode and Feeding Strategies

Along with the optimization of operational parameters, selection of fermentation mode also influences biocatalyst development and PHA synthesis. In particular, batch, fed-batch, and continuous fermentation modes, have been extensively studied in VFAs-PHA investigations. In batch reactors, C and nutrient source are provided at the same time but sometimes it is insufficient for bacterial growth and PHA accumulation. To avoid this, the fed-batch fermentation mode has been investigated by many researchers [68] by regulating intermittent substrate feeding.

It was also reported that during fed-batch fermentation, higher utilization of dissolved oxygen takes place which induces PHA accumulation [69]. Zhao et al. [70] investigated the production of PHBV and cell growth by employing fermentation in batch and fed-batch mode. The results showed that in fed-batch mode, PHBV and CDW yield were approximately 14 g/L and 29 g/L, respectively, which is four and eight times higher than the yield obtained in batch fermentation. In another study, the effects of pH and feeding regimes on PHA synthesis were assessed by employing VFAs generated after fermentation of a mixture of municipal wastewater and food waste [71]. The results revealed that feeding regimes directly influence PHA accumulation and rate of PHA synthesis. Other examples of a fermentation approach (batch and fed-batch) for the conversion of VFAs to PHA with pure and mixed microbial culture are presented in Table 3 and Table 4. A continuous fermentation strategy was found to be effective for maintaining stable nutrient conditions which led to the attainment of substantial PHA productivity and bacterial growth in a rapid and concomitant way which reduced PHA production cost [72]. However, implementation of continuous fermentation for commercial PHA production has not been thoroughly explored because of the possibility of loss of C source and contamination during continuous feeding which increases the overall production cost [73].

In addition, feeding of the substrate in different ways can directly influence microbial growth, PHA composition, and PHA accumulation. Pulse, stepwise, and continuous feeding regimes during fermentation have been widely studied to attain higher PHA accumulation [73,74]. In one study, an intermittent feeding approach was applied for the synthesis of P(3HB) by *Cupriavidus necator* utilizing VFAs generated after the fermentation of kitchen leftovers. In this study, PHB production under intermittent feeding was four times higher than PHB production attained in fed-batch culture (Omar et al., 2011). A “feast and famine” feeding regime is another prominent and effective approach that has been found effective to attain maximum PHA productivity and microbial growth. During the feast phase of fermentation, excess C is supplied, which enhances biocatalyst growth and stored excess C source in PHA granules, whereas, in the famine stage, there is no supply of additional C source which leads to utilization of reserve carbon sources for growth. Morgan-Sagastume et al. [75] evaluated the feasibility of PHA production by means of mixed culture under a feast and famine fermentation regime and it was observed that there was an increase of 34% in PHA accumulation (g PHA g VSS^−1^) relative to batch fermentation mode.

### 6.3. Using Mixed Microbial Culture

Mixed microbial cultures (MMC) are widely exploited in PHA synthesis relative to pure cultures because of certain advantages, including less maintenance requirements, lack of a requirement for an aseptic environment, less capital and lower operation costs [76,77]. Moreover, the stability and robustness of MMC counteracts internal and external distresses during the utilization of complex substrates and makes the PHA bioproduction process economically feasible and practically applicable [78]. The literature review related to the efficient transformation of VFAs to PHA, and its development and fermentation parameters, are shown in Table 4. Studies have been carried out concerning these factors so that scale up of PHB processes can be readily understood. However, there are certain limitations to the utilization of MMC for designing and developing commercial production methods for PHA using VFA waste streams. It is difficult to understand the precise roles and inclusive interrelationships among the microorganisms of MMC in the fermentation process. Intensive research is still needed to understand the microbial interactions in MMC to develop the process. In addition, during MMC fermentation, it is difficult to maintain consistency of microbial concentrations and to control the optimal stability of the microorganisms of MMC. Moreover, employing genetic engineering and synthetic biology methods to improve efficient MMC is at an infant stage and presents many challenges, particularly in horizontal gene transfer and sustaining homeostasis of MMC.

### 6.4. Genetic Engineering Approaches for Sustainable PHA Production Using VFAs

Improvement of the microbial strain by employing genetic engineering and molecular tools offers many advantages including improved carbon utilization and enhanced PHA production and quality. Application of these tools can be effective to reduce downstream bioprocesses and ultimately decreases the overall process cost. Hence, innovative research from biotechnology can aid in obtaining high cell biomass yield with high PHA output in large scale production. At the industrial scale, the polymers’ physical, chemical, thermal and elasticity properties are important and can be achieved by genetic engineering and optimizing other important fermentation conditions [90]. Genetic modifications can control PHA composition; the monomer composition directly impacts on the physical and mechanical properties of the bioplastics after fermentation. Recently, some investigators have studied genetically modified strains designed for the synthesis of co-polymer P(3HB-co-3HV) instead of homopolymer P3HB. Due to its crystalline structure, PHB has some limitations in its practical applications and P(3HB-co-3HV) is a better option for commercial applications [91,92]. In another study, Tran and Charles, [93], engineered Type II PhaC1 synthase enzyme from native *P. putida* which can assimilate a wider range of substrates and synthesizes medium chain length 3HA monomers. Related important examples of genetically modified strains and their PHA synthesis utilizing VFAs as a carbon substrate are presented in Table 5. Moreover, genomics, proteomics and metabolic engineering approaches could be useful for the development of desired metabolic pathways for the competent consumption of VFAs and improved PHA synthesis.

## 7. Conclusions

Extensive exploitation of conventional plastics is a worldwide threat to our ecosystems. The global bio-plastics market shows tremendous growth potential in producing sustainable products for diverse applications. Despite this, the production costs of biopolymers restrict the commercialization of PHA. The production of PHA using acidogenic effluents (VFAs) may be beneficial in terms of process cost, waste reduction, and environmental friendliness. This review has highlighted recent advances in the efficient bioconversion of VFAs to PHA and their existing challenges with the purpose of substitution for synthetic plastics. The review could be pivotal to the prospect of implementation of this technology for commercial bio-manufacturing.

However, many challenges still need to be addressed for effective VFAs-PHA production, as follows:

(1) Optimization of process parameters to generate VFAs in good quantity in the fermented waste. More research is needed for the cost effective separation of VFAs from acidogenic effluents. A further obstacle is the presence of ammonium and phosphorous in the VFA-rich acidogenic effluents.
(2) Extensive research and development is needed for the isolation of effective microbial strains which can tolerate VFAs and can effectively convert them into PHA with a high production rate.
(3) A major focus should be the effective downstream processing for the isolation of PHA with higher productivity and purity. The process should be cost effective and eco-friendly by avoiding input of harsh chemicals.
(4) It is a necessity to develop effective microbial strains by applying genetic engineering and molecular tools that can lead to efficient VFAs utilisation, enhanced biomass yield, and PHA productivity.
(5) Significant attention should be devoted to PHA studies at pilot and commercial scales to understand the molecular and engineering issues.

## Figures and Tables

**Table 1:** Chemical properties, their market size and usage of volatile fatty acids (adapted from [17,18,19,20]).

Volatile Fatty Acid	Chemical Structure and Formula	Chemical Properties	Production Methods	Global Market Size and CAGR	Usage/Application
Acetic acid	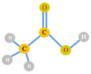 CH_3_COOH	MW: 60.05 Density:1.05 pKa: 4.76	Methanol carbonylation, oxidation of acetaldehyde and ethylene, oxidative and anaerobic fermentation	USD 9.3 billion in 2020; CAGR of 5.2%	Vinyl acetate monomer as adhesives, dyes, food additives, vinegar, ester manufacture
Butyric acid	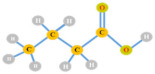 CH_3_ (CH_2_)_2_COOH	MW: 88.11 Density:0.96 pKa: 4.82	Oxidation of butyraldehyde, extraction from butter, anaerobic fermentation	USD 175 million in 2020; CAGR of 13.2%	Food additives (animal, human), chemical precursors, solvents, flavouring agents
Propionic acid	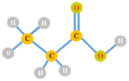 CH_3_CH_2_COOH	MW:74.08 Density:0.99 pKa: 4.88	Hydrocarboxylation of ethylene, aerobic oxidation of propionaldehyde, direct oxidation of hydrocarbons, anaerobic fermentation	USD 1.53 billion in 2020; CAGR of 2.7%	Food additives, flavoring, pharmaceuticals, animal feed supplements, fishing bait additives
Lactic acid	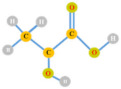 CH_3_CHOHCOOH	MW:90.08 Density:1.20 pKa:3.86	Chemical synthesis, anaerobic fermentation	USD 2.7 billion in 2020, CAGR of 8.0%	Polymers (polylactic acid) production, food products, additives, cleaning products
Valeric acid	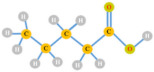 CH_3_(CH_2_)_3_COOH	MW:102.13 Density:0.93 pKa: 4.84	Oxo process from 1-butene and syngas, anaerobic fermentation	USD 15.06 billion in 2020; CAGR of 5.3%	Food additives, in perfumes, cosmetics, and foodstuffs

MW = molecular weight (g/mol); Density = (g/cm^3^); CAGR = compound annual growth rate.

**Table 2:** Worldwide known commercial polyhydroxyalkanoate (PHA) manufacturing plants utilizing various carbon sources.

Company	Carbon Source	Type of PHA	Final PHA (% CDW)	Production (Tones/Annum)	Year	Potential Uses
Tianjin Northern Food, Tianjin, China	Glucose	PHB	>80%	Pilot scale	1990s	Raw materials
Chemie Linz, btf, Linz, Austria Biomers, Ulm, Germany	Glucose or sucrose	PHB	>75%	20–100	1980s	Packaging and drug delivery
Jiangsu LanTian, Taizhou, China	Glucose	PHB	>80%	20–100	1990s	Packaging and drug delivery
ICI, UK Zhejiang Tianan, Hangzhou, China	Glucose + propionate	PHBV	>75%	300– 2000	1980s to 1990s 1990s to present	Packaging and raw materials
Metabolix, Woburn, MA, USA	Glucose +	P3HB4HB	>75%	Unknown	1980s to present	Packaging
P&G, Kaneka, Osaka, Japan	Fatty acids	PHBHHx	>80%	Unknown	1990s to present	Packaging
P&G, Jiangmen Biotech Ctr, Jiangmen, China	Lauric acid	PHBHHx	<50%	Unknown	1990s	Raw materials
Shandong Lukang, Jining, China	Lauric acid	PHBHHx	>50%	Pilot scale	2005 to present	Raw materials and packaging
ETH, Zürich, Switzerland	Fatty acids	MCL PHA	>60%	NA		Raw materials and packaging
Biocycles, São Paulo, Brazil	Sucrose	PHB	>50%	100	1990s to present	Raw materials

CDW: Cell dry weight.

**Table 3:** PHA production using individual and mixed VFAs by pure microbial culture.

**Employed Substrate**	Microorganism	Fermentation Type	DCW (g/L)	PHA Accumulation (%)	PHA Yield (g/L)	Type of PHA	Reference
Acetic acid	*Ralstonia eutropha* ATCC 17699	Shaking flask	5.4	30.8	1.66	P(3HB)	[37]
Propionic acid	*Ralstonia eutropha* ATCC 17699	Shaking flask	14.0	29.3	4.10	P(3HB)	[37]
Butyric acid	*Ralstonia eutropha* ATCC 17699	Shaking flask	14.5	31.9	4.62	P(3HB)	[37]
Propionic acid + glucose Propionic acid + glucose Acetic acid + propionic acid + glucose	*Pseudomonas* sp. ST2 *Bacillus* sp. CS8 *Pseudomonas* sp. ST2 + *Bacillus* sp. CS8	Shaking flask	ND	34.0 24.0 35.0	ND	P(3HB-co-3HV)	[38]
Acetic acid Propionic acid Butyric acid Valeric acid	*Methylocystis hirsuta* DSM 18500	Shaking flask	ND	2.4 1.1 1.8 9.0	ND	P(3HB-co-3HV)	[39]
Acetic acid + biogas Propionic acid + biogas Butyric acid + biogas Valeric acid + biogas	*Methylocystis hirsuta* DSM 18500	Shaking flask	ND	52.3 47.9 52.2 53.8	ND	P(3HB) P(3HB-co-3HV) P(3HB) P(3HB-co-3HV)	[39]
Acetic acid	*Ralstonia eutropha* H16 (DSM 428)	Shaking flask	ND	33.3	0.5	P(3HB)	[46]
Acetic acid	*Clostridium* *autoethanogenum*	Fed-batch	ND	24	ND	P(3HB)	[47]
Acetic acid + propionic acid + butyric acid	*Ralstonia eutropha* ATCC 17699	Batch	1.2	25.0	0.30	P(3HB-co-3HV)	[41]
Acetic acid + propionic acid + butyric acid	*Ralstonia eutropha* KCTC 2658	Batch	1.5	50	0.75	P(3HB-co-3HV)	[41]
Acetic acid Acetic acid Acetic acid Valeric acid	*Corynebacterium hydrocarboxydans* *Nocardia lucida* *Rhodococcus* sp. NCIMB 40126	Batch	ND	21.0 20.0 29.0 43.0	ND	P(3HB-co-3HV) P(3HB-co-3HV) P(3HB-co-3HV) P(3HB-co-3HV)	[48]
Butyric acid Valeric acid	*Bacillus* sp. INT005	Shaking flask	0.8 0.7	31.5 18.8	0.25 0.13	P(3HB) P(3HB-co-3HV)	[49]
Propionic acid + glucose Propionic acid + glycerol	*Bacillus megaterium* OU303A	Shaking flask	ND	62.4 57.2	ND	P(3HB)	[50]
Butyric acid + valeric acid + Tween 20	*Haloferax mediterranei*	Fed batch	ND	58.9	ND	P(3HB-co-3HV)	[51]
Olive mill wastewater effluent rich in acetic, propionic, and butyric acid	*Cupriavidus necator*	Two-stage batch cultivation	2.0	55.0	1.1	P(3HB-co-3HV)	[52]
Acetic acid 0.5 g/L 5.0 g/L	*Bacillus cereus* strain HY	Shaking flask	1.82 2.94	40.2 49.8	0.73 1.46	P(3HB)	[35]
Acetic, propionic, butyric	*Alcaligenes eutrophus*	Shaking flask	6.64	86.5	5.75	P(3HB)	[53]
Acetic, propionic and n-butyric acids.	Comamonas sp. EB172	Fed-batch	7.2	90	6.48	P(3HB-co-3HV)	[54]
Acetic, propionic and n-butyric acids.	*Comamonas* sp. EB172	Fed-batch	9.8	59	5.78	P(3HB-co-3HV)	[55]
Acetic, propionic and n-butyric acids.	*Comamonas* sp. EB172	Shake flask	3	20	0.6	P(3HB-co-3HV)	[56]
Acetic, propionic, butyric	*Thauera* sp. *Paracoccus denitrificans*	Sequencing batch reactor	ND	34.2	227.8 mg/L 673 mg/L	P(3HB) P(3HB-co-3HV)	[57]
Propionic and butyric acid	*Ralstonia eutropha*	Batch	1.53	46.5	0.7	P(3HB)	[58]
Lactic acid and acetic acid	*Ralstonia eutropha*	Fed batch	75	73.1	54.8	P(3HB)	[59]
Lactic acid and acetic acid	*Cupriavidus necator* CGUG 52238	Batch	ND	84.54 (*w*/*w*)	0.79 g/g	P(3HB)	[60]

**Table 4:** PHA production employing VFAs comprising waste streams by mixed microbial culture.

Employed Substrate	Inoculum Source	Fermentation Type	PHA Accumulation (%)	PHA Yield (g/L)	Type of PHA	Reference
Fermented molasses	Mixed activated sludge culture	Sequencing batch reactor	66.0	ND	P(3HB-co-3HV)	[3]
Acidogenic effluent	Enriched mixed cultures	Batch	54	ND	P(3HB-co-3HV)	[31]
Fermented food waste + dewatered sludge	Mixed activated sludge culture	Batch	64.5	ND	P(3HB-co-3HV)	[71]
Acetic acid	Mixed activated sludge culture	Sequencing batch reactor	40.0	ND	P(3HB)	[79]
Acetic acid	Mixed activated sludge culture	Batch reactor	78.5	5–180 C_mmol_/l for acetate	P(3HB)	[80]
Acetic acid	Mixed microbial culture	Acetate-fed sequencing batch reactor	89.0	ND	P(3HB)	[81]
Municipal wastewater + acetic acid	Mixed activated sludge culture	Sequencing batch reactors	30.0	28 mg C/g SS/h	P(3HB)	[82]
Fermented paper mill wastewater	Mixed activated sludge culture	Batch	48.0	0.11 kg of PHA/kg of COD (treated influent)	P(3HB-co-3HV)	[83]
Fermented paperboard mill wastewater	Mixed microbial culture	Sequencing batch reactors	67.4	0.46 ± 0.09 C_-mol_ C_-mol−1_	P(3HB-co-3HV)	[84]
Sludge hydrolysis liquid	Heat pretreated waste sludge	Sequencing batch reactor	24.1	0.46 mg COD/mg COD	P(3HB-co-3HV)	[85]
Fermented crude glycerol	Mixture of equivalent ratio of anaerobic sludge and aerobic sludge	Sequencing batch reactor	76.0	0.84 g COD PHA/g COD S	P(3HB-co-3HV)	[86]
Fermented wood waste	Acidogenic sludge	Batch	50.3	0.71 g COD PHA/g COD VFAs	P(3HB-co-3HV)	[87]
Fermented cheese whey	Phototrophic mixed cultures	Sequencing batch reactors	30.0	0.83 ± 0.07 C_mol-_PHB/C_mol-Acet_	P(3HB-co-3HV)	[88]
Fermented Food waste	Acidogenic mixed bacteria	Batch	23.7	0.168 g PHA_COD_/g WW_COD_	P(3HB-co-3HV)	[66]
Fermented Food waste	Industrial wastewater	Fed-batch	39.6	ND	P(3HB-co-3HV)	[89]

**Table 5:** PHA production using VFAs as a carbon source by employing genetically modified microbial culture.

Employed Substrate	Microorganism	Fermentation Type	PHA Yield (g/L)	Type of PHA	Reference
Propionic acid	*Herbaspirillum* *seropedicae* Z69 2-methylcitrate synthase (PrpC) gene	Shaking flask	3HV yield 0.80 g/g	PHBV	[19]
Acetic acid	*Bacillus cereus* strain HY-3	Shaking flask	PHB content—49.2%	PHB	[35]
Glycerol propionic acid	*Salmonella enterica* serovar Typhimurium	Shake flask	3HV yield 19.4 (mol%)	P(3HB-co-3HV)	[64]
Acetate	*Escherichia coli* Overexpressed pta-ackA and acs genes	Shaking flask	1.27 g/L	P3HB	[94]
Glycerol	E. XL1-Blue filamentation-suppressed FtsZ	Fed-batch	149 g/L	PHB	[95]
Glycerol + propionic acid	*S. enterica* propionyl-CoA prpC, as a host	Shaking flask	34.2 ± 15 (%DCW)	PHBV	[96]
Propionic acid	*Burkholderia* sp. IPT 101 propionyl- *prp* mutants	Batch	Y_3HV:Prop_ 1.20 g g^−1^	PHBV	[97]
Glucose propionate	Recombinant *E. coli* XL10-Gold/pBHR68	Fed-batch	3HV yield 0.31 g/g	P(3HB-co-3HV)	[98]
Glucose propionate	Recombinant *E. coli* XL10-Gold/pBHR68-prpP	Fed-batch	3HV yield 0.46 g/g	P(3HB-co-3HV)	[98]
